# Global Discrepancies between Numbers of Available SARS-CoV-2 Genomes and Human Development Indexes at Country Scales

**DOI:** 10.3390/v13050775

**Published:** 2021-04-28

**Authors:** Philippe Colson, Didier Raoult

**Affiliations:** 1 IHU Méditerranée Infection, 19–21 Boulevard Jean Moulin, 13005 Marseille, France; philippe.colson@univ-amu.fr; 2 Aix-Marseille University, Institut de Recherche pour le Développement (IRD), Assistance Publique-Hôpitaux de Marseille (AP-HM), MEPHI, 27 Boulevard Jean Moulin, 13005 Marseille, France

**Keywords:** SARS-CoV-2, genome, next-generation sequencing, country-scale development, world, variant

## Abstract

It has now been over a year since SARS-CoV-2 first emerged in China, in December 2019, and it has spread rapidly around the world. Some variants are currently considered of great concern. We aimed to analyze the numbers of SARS-CoV-2 genome sequences obtained in different countries worldwide until January 2021. On 28 January 2021, we downloaded the deposited genome sequence origin from the GISAID database, and from the “Our world in data” website we downloaded numbers of SARS-CoV-2-diagnosed cases, numbers of SARS-CoV-2-associated deaths, population size, life expectancy, gross domestic product (GDP) per capita, and human development index per country. Files were merged and data were analyzed using Microsoft Excel software. A total of 450,968 SARS-CoV-2 genomes originating from 135 countries on the 5 continents were available. When considering the 19 countries for which the number of genomes per 100 deaths was >100, six were in Europe, while eight were in Asia, three were in Oceania and two were in Africa. Six (30%) of these countries are beyond rank 75, regarding the human development index and four (20%) are beyond rank 80 regarding GDP per capita. Moreover, the comparisons of the number of genomes sequenced per 100 deaths to the human development index by country show that some Western European countries have released similar or lower numbers of genomes than many African or Asian countries with a lower human development index. Previous data highlight great discrepancies between the numbers of available SARS-CoV-2 genomes per 100 cases and deaths and the ranking of countries regarding wealth and development.

## 1. Introduction

The SARS-CoV-2 pandemic, which has been spreading for almost a year, has generated considerable global efforts in the sequencing, collection, and analysis of viral genomes. Sequence databases and various tools for storing, downloading, classifying, and analyzing these genomes have quickly become available [1,2]. In particular, GISAID sequence database hosts a collection of SARS-CoV-2 genomic sequences obtained worldwide (https://www.gisaid.org/; accessed on 28 January 2021) [1]. Our team has produced a large number of genome sequences for SARS-CoV-2, in particular when the incidence of cases considerably re-increased during the summer [3,4,5,6,7]. This enabled us to point out the existence of variants very early (which are strains that differ from all others by a set of several mutations and have reached a detectable population size) during the summer of 2020 [4]; we named those identified in our institute Marseille-1 to Marseille-10. They have been responsible for successive or overlapping epidemics, before becoming established at our country’s scale.

Currently, the emergence and spread of SARS-CoV-2 genotypic features are in the spotlight. Firstly, different viral variants that have emerged appear to be associated with different epidemic dynamics and clinical severities. What we observed for the first SARS-CoV-2 variant that we had identified in July 2020, which originated from the African continent and was named “Marseille-1” [5], has reproduced with the Marseille-4 variant [7] (also known as clade 20A.EU2 [8]), and is currently observed with the UK (20I/501Y.V1), South African (20I/501Y.V1), and Brazilian (20I/501Y.V1) variants. Thus, some of these variants have either demonstrated or they are suspected to have greater transmissibilities and have become the predominant strains nationwide, and some were reported to cause diseases with different severities [5,7,9,10,11,12]. In addition, the majority of these viral variants harbor amino acid changes in the viral spike, the protein that enables virus entry into human cells through binding to the ACE2 receptor, which is also the main target of neutralizing antibodies elicited by natural infection or vaccine immunization [13,14,15]. Accordingly, changes in the spike amino acid sequence of these variants have been reported to increase viral binding to the ACE2 receptor and to allow virus escape from neutralizing antibodies induced by prior infection or vaccine immunization [15,16,17]. Moreover, therapies such as remdesivir, convalescent plasma or cocktails of anti-spike antibodies, particularly in immunocompromised patients, could increase the mutation rate of SARS-CoV-2 genomes and have been associated with the rapid occurrence of several amino acid changes within the spike [14,18,19,20]. Among these amino acid changes, some are present in the UK, South African, and Brazilian variants. Thus, it should have been necessary, in the cases of absence of viral clearance after administration of these treatments, to systematically sequence SARS-CoV-2 genomes and check for the occurrence of mutations. For example, this should have been performed in Hueso et al.’s study for the five patients who were not cleared of the virus after convalescent plasmatherapy in order to determine whether mutations located in the spike had not been selected by the transfused antibodies [21].In addition, cases are increasingly being reported of patients who experienced a second infection with SARS-CoV-2 several months after a first infection that was followed by viral clearance [22,23,24,25]. In our institute, two successive infections with different variants have been observed to date in nearly fifty patients [25]. Systematic sequencing of the genomes of the viruses involved in the two distinct infections is essential to understanding which viral strains can resist, through their mutation patterns, immune responses elicited by a first infection with a distinct strain. Finally, in the current setting of massive vaccine strategies that in Western countries are based on the spike protein, it is absolutely critical to analyze the viral genomes in all cases of vaccine failures in order to determine which viral mutants and variants are involved. In our country, for example, the majority of SARS-CoV-2 strains that are currently circulating have a spike protein whose amino acid sequence differs from that used in vaccines, which corresponds to strains that no longer exist or are in the minority [6,7] (https://nextstrain.org/groups/neherlab/ncov/france; accessed on 11 April 2021). Under these conditions, the question of the impact of some variants on the level of protection conferred by prior infection or vaccine immunization arises [17,26,27,28].

An earlier study analyzed the number of SARS-CoV-2 genomes per reported COVID-19 case nationwide, based on the sequences available in the GISAID database in early September 2020. It pointed out substantial differences between countries worldwide, including between those on the same continent as well as the good level of sequencing efforts of some low and middle-income countries [29]. Here, we wanted to analyze the numbers of genome sequences of SARS-CoV-2 obtained in the different countries worldwide by the end of January 2021, and to correlate them to the numbers of SARS-CoV-2 cases and SARS-CoV-2-associated deaths and to the wealth and investment in health of these countries.

## 2. Materials and Methods

On 28 January 2021, we downloaded the nextmeta file that contains the origin of deposited genome sequences from the GISAID database (https://www.gisaid.org/; accessed on 28 January 2021) [1]). On the same day, we also downloaded from the “Our world in data” website (https://ourworldindata.org/; accessed on 28 January 2021) the numbers of SARS-CoV-2-diagnosed cases and SARS-CoV-2-associated deaths per country as well as various epidemiological data, including population size, life expectancy, gross domestic product (GDP) per capita, and human development index (collected from URL: https://covid.ourworldindata.org/data/owid-covid-data.xlsx; accessed on 11 April 2021). According to the United Nations Development Programme (http://hdr.undp.org/en/content/human-development-index-hdi; accessed on 11 April 2021), the human development index is the geometric mean of normalized indices for the health dimension (assessed by life expectancy at birth), the education dimension (assessed by mean of years of schooling for adults ≥ 25 years of age, and expected years of schooling for children of school-entering age), and the standard of living dimension (assessed by gross national income per capita). This index was used as a measure of country development to figure out if this latter was related to the capacity and/or willingness to perform next-generation sequencing to assess SARS-CoV-2 genomic epidemiology. Files were merged and data were analyzed using Microsoft Excel software (https://www.microsoft.com; accessed on 11 April 2021). We standardized the numbers of genomes sequenced per 100 SARS-CoV-2-diagnosed cases and per 100 SARS-CoV-2-associated deaths. Data were plotted using Microsoft Excel and GraphPad Prism v.5 (https://www.graphpad.com; accessed on 11 April 2021) software. The numbers of genomes per country taken into account were those released by a given country regardless of whether sequencing was performed inside or outside this country, considering the origin of the clinical specimen. We also checked the numbers of SARS-CoV-2 genomes for some countries on other sequence databases including the National Center for Biotechnology Information (NCBI; https://www.ncbi.nlm.nih.gov/; accessed on 11 April 2021), the European Bioinformatics Institute (EMBL-EBI; https://covid-19.ensembl.org/index.html; accessed on 11 April 2021), and the China National Center for Bioinformation (CNCB; https://bigd.big.ac.cn/ncov/; accessed on 11 April 2021).

## 3. Results

A total of 450,968 SARS-CoV-2 genomes were available from the GISAID database on 28 January 2020. They originated from five continents, from 135 countries and 8919 laboratories. The mean (± standard deviation) number of genomes per country was 3340 ± 18,498 (range, 1–192,556) and the median number was 129. The mean number of genomes per 100 SARS-CoV-2-associated deaths per country was 270 ± 1422 (0.06–14,397) and the median was 6.2. Finally, the mean number of genomes per 100 SARS-CoV-2 diagnosed cases per country was 2198 ± 9105 (0.001–70) and the median number was 0.120.

**Figure 1:**
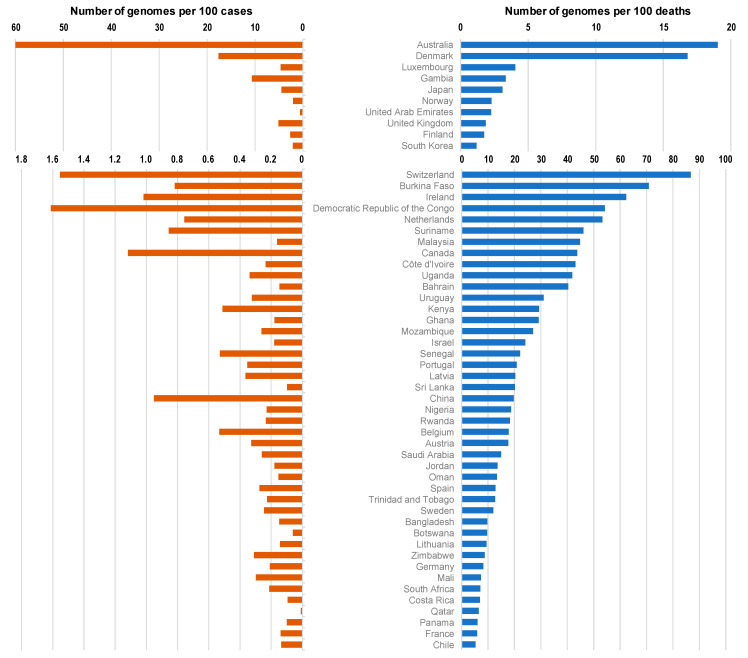
Numbers of SARS-CoV-2 genomes per 100 SARS-CoV-2-diagnosed cases (**left**, orange) and per 100 SARS-CoV-2-associated deaths (**right**, blue) according to countries.

**Figure 2:**
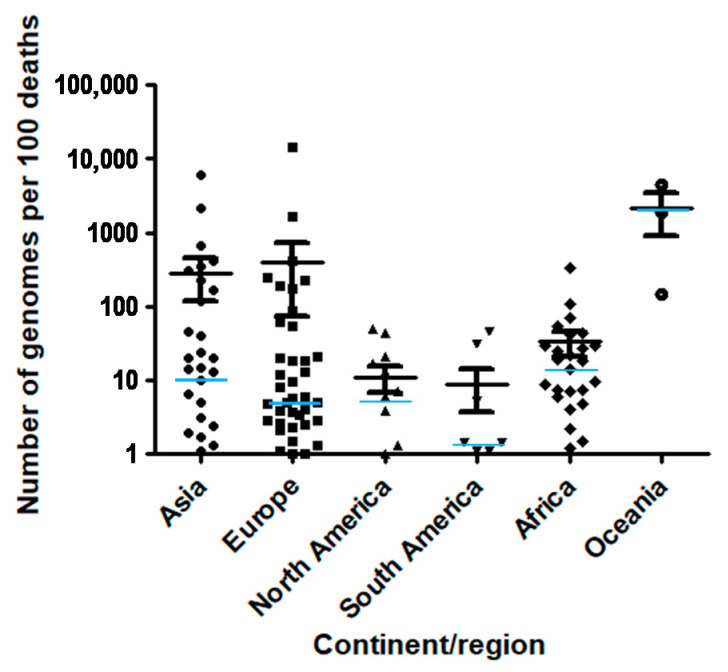
Number of SARS-CoV-2 genomes per 100 SARS-CoV-2-associated deaths according to continent/region. Median value for each region is indicated by a light blue horizontal bar.

The top 100 source laboratories accounted for 72% (n = 324,837) of available genomes (Supplementary Table S1). They were mostly located in the USA (62%; n = 24), in England (21), in Denmark (11), and in the Netherlands (6). When considering the 19 countries for which the number of genomes per 100 deaths was > 100, 6 were in Europe (Iceland (number of genomes per 100 deaths = 14,397), Denmark (1680), Luxembourg (405), Norway (229), UK (186), and Finland (174)), while 8 were in Asia (Singapore (5969), Taiwan (2143), Thailand (653), Vietnam (406), Mongolia (350), Japan (310), Brunei (167), and South Korea (117)), 3 were in Oceania (New Zealand (4380), Australia (1902), and Papua New Guinea (144)) and 2 were in Africa (Gambia (344), and Equatorial Guinea (110)) (Figure 1 and Figure 2; Table 1).

In addition, six (30%) of these countries have a human development index below the mean value for the 135 countries studied here (0.756): Thailand (human development index = 0.755), Vietnam (0.694), Mongolia (0.741), Gambia (0.460), Papua New Guinea (0.544), and Equatorial Guinea (0.591). Moreover, all these six countries have a GDP per capita below the mean value for the 135 countries studied here (22,884 US dollars) (Table 1). Similarly, when considering the 24 countries for which the number of genomes per 100 diagnosed cases was ≥ 1, eight were in Asia (Taiwan, Vietnam, Japan, Thailand, Singapore, Brunei, South Korea, and China) and three were in Africa (Gambia, Equatorial Guinea, and Democratic Republic of Congo). In addition, seven (29%) of these countries have a human development index below the mean value for the 135 countries studied here (0.756): Gambia (human development index = 0.460), Vietnam (0.694), Thailand (0.755), Equatorial Guinea (0.591), Democratic Republic of Congo (0.457), Papua New Guinea (0.544), and China (0.752), and all these seven countries have a GDP per capita below the mean value for the 135 countries studied here (22,884 US dollars) (Table 1). 

**Figure 3:**
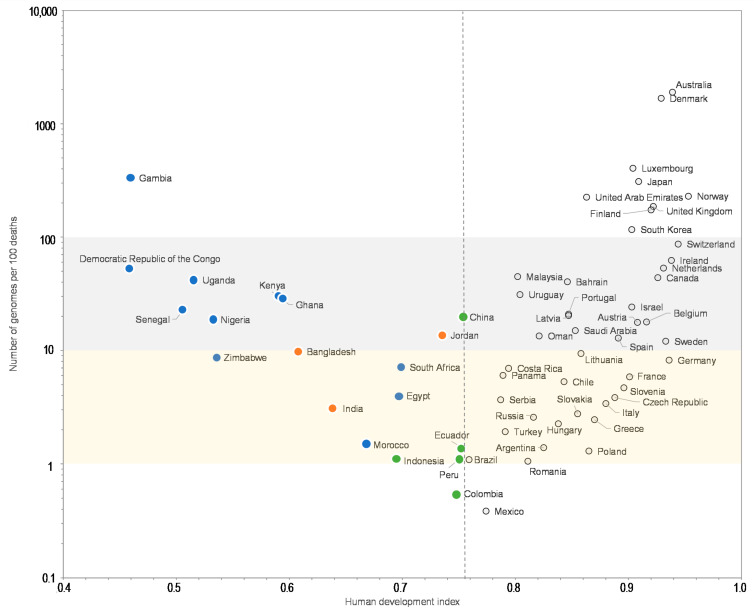
Number of SARS-CoV-2 genomes per 100 SARS-CoV-2-associated deaths vs. human development index.

**Figure 4:**
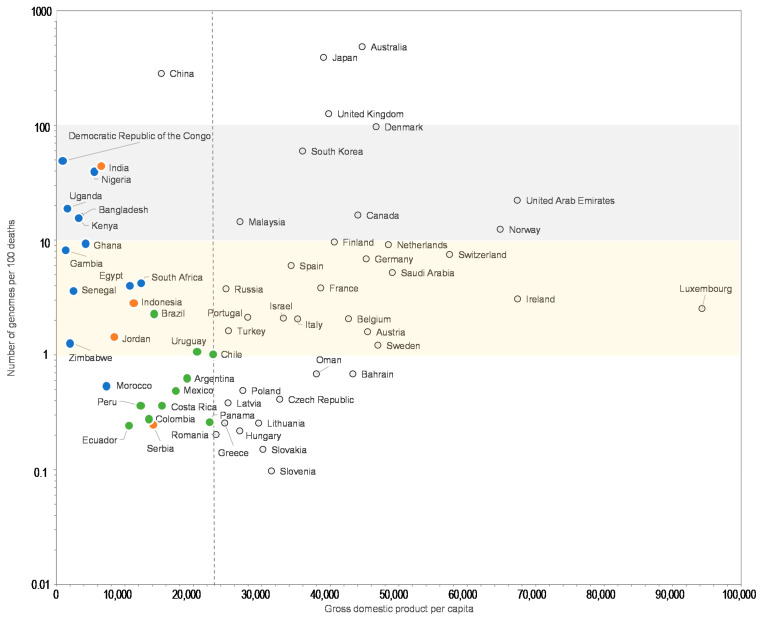
Number of SARS-CoV-2 genomes per 100 SARS-CoV-2-associated deaths vs. gross domestic product per capita (GDP).

Moreover, the comparisons of the number of SARS-CoV-2 genomes sequenced per 100 SARS-CoV-2-associated deaths and the human development index by country show that some Western European countries such as Germany (8.2 genomes per 100 deaths; human development index = 0.936), France (5.9; 0.901), or Italy (3.4; 0.880) have released similar or lower numbers of genomes than many African or Asian countries with a lower human development index, among which Egypt (4.0 genomes per 100 deaths; human development index = 0.696), Zimbabwe (8.7; 0.535), Nigeria (19; 0.532), Senegal (22; 0.505), Democratic Republic of Congo (54; 0.457), Gambia (334; 0.460), Bangladesh (9.8; 0.608), and China (20; 0.752) (Figure 3). Similar observations can be made when comparing the number of genomes sequenced per 100 deaths and the GPD per capita (Figure 4) by country. Finally, we checked for several countries that they did not submit significant numbers of SARS-CoV-2 genome sequences to sequence databases other than GISAID and particularly found a similar number of genomes in the China NBI sequence database that compiles sequences from GISAID and GenBank in comparison with GISAID alone (Supplementary Table S2).

For a better legibility of the graph, only countries with more than 100 SARS-CoV-2 genomes are shown. Grey and yellow strips indicate countries with numbers of genomes per 100 deaths between 10 and 100, and between 1 and 10, respectively. Blue, green, and orange dots mark countries from Africa, America, and other regions, respectively, with a human development index below the mean value for all 135 countries studied here (0.756).

For a better legibility of the graph, only countries with more than 100 SARS-CoV-2 genomes are shown. Grey and yellow strips indicate countries with numbers of genomes per 100 deaths between 10 and 100, and between 1 and 10, respectively. Blue, green, and orange dots mark countries from Africa, America and other regions, respectively, with a GDP per capita below the mean value for all 135 countries studied here (22,884 US dollars). GDP is in US dollars.

## 4. Discussion

This analysis, conducted 10 months after WHO declared COVID-19 a pandemic (https://www.who.int/director-general/speeches/detail/who-director-general-s-opening-remarks-at-the-media-briefing-on-covid-19, accessed on 11 march 2020), shows great disparities according to the country in the numbers of SARS-CoV-2 genomes available per 100 cases and deaths, as well as substantial discrepancies between these numbers and the ranking of countries based on their wealth and development, although this was not a general pattern. Here, we considered SARS-CoV-2 genomes from a given country regardless of whether they were obtained inside or outside this country. Therefore, the present analysis shows that several developed countries had either a technological or organizational delay in terms of high throughput sequencing, and/or an insufficient purposefulness to monitor SARS-CoV-2 genetic and proteic diversity and variability. Thus, firstly, in some developed countries, the importance of detecting, characterizing, and surveying SARS-CoV-2 variants may have been initially overlooked. Secondly, the majority of laboratories may have been unable to produce a large number of SARS-CoV-2 genomic sequences because the necessary infrastructure was not in place at the start of the pandemic. This includes the fact that these laboratories did not possess or even did not have access to next-generation sequencing instruments for clinical diagnosis, but only possessed sequencers using Sanger technology. Another reason could have been the lack of organization in terms of human resources or pre-existing training, allowing a high capacity for high-throughput sequencing. Other obstacles could have been global supply chain issues for reagents and consumables. In contrast, several developing countries exhibited wills as well as capacities to sequence SARS-CoV-2 genomes and scaled up next-sequencing technologies [30,31,32]. This is another example that the SARS-CoV-2 pandemic is reshuffling the cards globally.

Limitations to the present study are that it may not comprehensively take into account all SARS-CoV-2 genome sequences obtained in each country. Thus, all genomes sequenced may not be submitted to a sequence database. They may also be submitted to other sequence databases than GISAID, but we did not observe by screening four different sequence databases that these results were biased by disparities between the proportions of sequences submitted to GISAID and other major sequence databases according to countries. Moreover, the human development index and GPD per capita analyzed here do not necessarily reflect the strength of medical research and technology at a country scale.

Such a worldwide distribution of the availability of SARS-CoV-2 genomes as observed here is very interesting. Indeed, several issues related to SARS-CoV-2 genotypic features which are of paramount importance are currently in the forefront of the SARS-CoV-2 pandemic. SARS-CoV-2 variants cause successive or overlapping epidemics with various kinetics, levels of contribution to the total burden of SARS-CoV-2 infections and durations [5,6,7]. In addition, they can be associated with differences regarding disease transmissibility and severity, and they can have the potential to evade immune responses elicited by prior infection or vaccine immunization [5,7,16,17,26,27,28].

Overall, in a new disease caused by viruses with a high mutation rate, as we have learned for a long time with human immunodeficiency virus and hepatitis C virus, it is absolutely necessary to survey and monitor viral genome sequences to detect mutants and variants in order to identify possible differences in terms of transmissibility, clinical severity, resistance to treatments, and escape from vaccine immunity as well as natural immunity. SARS-CoV-2 genome-based surveillance should optimally be continuous with weekly assessments and should be capable of detecting the emergence of the viral variants and monitoring the dynamic and outcome of their epidemics. Considering previous data, broad-scale SARS-CoV-2 genomic surveillance should have been a priority for all developed countries that had the means to perform it.

## Figures and Tables

**Table 1:** Number of genomes, of SARS-CoV-2-diagnosed cases, of SARS-CoV-2-associated deaths per country, and various epidemiological data among which population size, life expectancy, gross domestic product (GDP) per capita, or human development index.

Country	Continent/Region	Number of Genomes	Number of Genomes per 100 Cases	Number of Genomes per 100 Deaths	Number of Cases	Number of Deaths	Population	GDP per Capita	Human Development Index
Iceland	Europe	4175	69.6	14,397	6001	29	341,25	46,483	0.935
Singapore	Asia	1731	2.9	5969	59,425	29	5,850,343	85,535	0.932
New Zealand	Oceania	1095	47.5	4380	2305	25	4,822,233	36,086	0.917
Taiwan	Asia	150	16.8	2143	895	7	23,816,775	-	-
Australia	Oceania	17,29	60.0	1902	28,799	909	25,499,881	44,649	0.939
Denmark	Europe	34,819	17.6	1680	197,892	2072	5,792,203	46,683	0.929
Thailand	Asia	496	3.1	653	16,221	76	69,799,978	16,278	0.755
Vietnam	Asia	142	8.6	406	1651	35	97,338,583	6172	0.694
Luxembourg	Europe	2325	4.6	405	50,228	574	625,976	94,278	0.904
Mongolia	Asia	7	0.4	350	1,71	2	3,278,292	11,841	0.741
Gambia	Africa	427	10.6	334	4019	128	2,416,664	1562	0.460
Japan	Asia	17,052	4.5	310	380,644	5503	126,476,458	39,002	0.909
IHU Méditerranée Infection *	*Europe*	*1585*	*5.2*	*250*	*30,237*	*633*	*-*	*-*	*-*
Norway	Europe	1278	2.1	229	62,276	557	5,421,242	64,8	0.953
United Arab Emirates	Asia	1845	0.6	225	293,052	819	9,890,400	67,293	0.863
United Kingdom	Europe	192,556	5.1	186	3,754,448	103,324	67,886,004	39,753	0.922
Finland	Europe	1154	2.6	174	44,039	664	5,540,718	40,586	0.920
Brunei	Asia	5	2.8	167	180	3	437,483	71,809	0.853
Papua New Guinea	Oceania	13	1.5	144	851	9	8947,027	3823	0.544
South Korea	Asia	1631	2.1	117	77,395	1399	51,269,183	35,938	0.903
Equatorial Guinea	Africa	95	1.7	110	5492	86	1,402,985	22,605	0.591
Switzerland	Europe	8071	1.6	87	519,404	9308	8,654,618	57,41	0.944
Burkina Faso	Africa	85	0.8	71	10,377	120	20,903,278	1703	0.423
Ireland	Europe	1973	1.0	62	193,645	3167	4,937,796	67,335	0.938
Democratic Republic of Congo	Africa	360	1.6	54	22,322	665	89,561,404	808	0.457
Netherlands	Europe	7422	0.8	53	979,702	13,925	17,134,873	48,473	0.931
Saint Vincent and the Grenadines	North America	1	0.1	50	827	2	110,947	10,727	0.723
Surinam	South America	71	0.9	46	8293	154	586,634	13,767	0.720
Malaysia	Asia	321	0.2	45	198,208	717	32,365,998	26,808	0.802
Canada	North America	8613	1.1	44	770,433	19,659	37,742,157	44,018	0.926
Côte d’Ivoire	Africa	65	0.2	43	27,694	151	26,378,275	3601	0.492
Uganda	Africa	133	0.3	42	39,424	318	45,741,000	1698	0.516
Bahrain	Asia	150	0.1	40	101,503	372	1,701,583	43,291	0.846
Uruguay	South America	129	0.3	31	39,887	415	3,473,727	20,551	0.804
Kenya	Africa	514	0.5	29	100,422	1753	53,771,300	2993	0.590
Ghana	Africa	114	0.2	29	63,883	390	31,072,945	4228	0.592
Mozambique	Africa	94	0.3	27	35,833	347	31,255,435	1136	0.437
Benin	Africa	12	0.3	25	3786	48	12,123,198	2064	0.515
Israel	Asia	1128	0.2	24	628,895	4669	8,655,541	33,132	0.903
Senegal	Africa	136	0.5	22	25,711	614	16,743,930	2471	0.505
Portugal	Europe	2422	0.4	21	685,383	11,608	10,196,707	27,937	0.847
USA	North America	89,814	0.3	21	25,766,681	433,196	331,002,647	54,225	0.924
Latvia	Europe	233	0.4	20	63,992	1148	1,886,202	25,064	0.847
Sri Lanka	Asia	60	0.1	20	61,586	297	21,413,250	11,669	0.770
China	Asia	949	1.0	20	99,746	4813	1,439,323,774	15,309	0.752
Nigeria	Africa	290	0.2	19	127,024	1547	206,139,587	5338	0.532
Rwanda	Africa	34	0.2	18	14,529	186	12,952,209	1854	0.524
Belgium	Europe	3743	0.5	18	702437	20,982	11,589,616	42,659	0.916
Austria	Europe	1344	0.3	18	410,23	7607	9,006,400	45,437	0.908
Antigua and Barbuda	North America	1	0.5	17	215	6	97,928	21,491	0.780
Saudi Arabia	Asia	953	0.3	15	367,276	6366	34,813,867	49,045	0.853
Sierra Leone	Africa	11	0.3	14	3,282	77	7,976,985	1390	0.419
Jordan	Asia	581	0.2	14	324,169	4269	10,203,140	8337	0.735
Oman	Asia	205	0.2	13	133,728	1527	5,106,622	37,961	0.821
Spain	Europe	7431	0.3	13	2,705,001	57,806	46,754,783	34,272	0.891
Trinidad and Tobago	North America	17	0.2	13	7,52	134	1,399,491	28,763	0.784
Sweden	Europe	1388	0.2	12	564,557	11,52	10,099,270	46,949	0.933
Bangladesh	Asia	792	0.1	9.8	533,953	8087	164,689,383	3524	0.608
Botswana	Africa	13	0.1	9.7	21,293	134	2,351,625	15,807	0.717
Lithuania	Europe	258	0.1	9.4	180,16	2749	2,722,291	29,524	0.858
Zimbabwe	Africa	101	0.3	8.7	32,646	1160	14,862,927	1900	0.535
Germany	Europe	4582	0.2	8.2	2,194,562	55,883	83,783,945	45,229	0.936
Mali	Africa	24	0.3	7.3	8056	328	20,250,834	2014	0.427
Guinea	Africa	6	0.0	7.3	14,435	82	13,132,792	1999	0.459
South Africa	Africa	3062	0.2	7.1	1,437,798	43,105	59,308,690	12,295	0.699
Costa Rica	North America	181	0.1	7.0	192,637	2599	5,094,114	15,525	0.794
Qatar	Asia	16	0.0	6.5	150,28	248	2,881,060	116,936	0.856
Panama	North America	314	0.1	6.0	316,808	5196	4,314,768	22,267	0.789
Gabon	Africa	4	0.0	5.9	10,536	68	2,225,728	16,562	0.702
France	Europe	4379	0.1	5.9	3,166,145	74,601	65,273,512	38,606	0.901
Liechtenstein	Europe	3	0.1	5.8	2475	52	38,137	-	0.916
Chile	South America	966	0.1	5.3	714,143	18,174	19,116,209	22,767	0.843
Malta	Europe	13	0.1	5.0	17,4	261	441,539	36,513	0.878
Estonia	Europe	20	0.0	4.9	42,656	406	1,326,539	29,481	0.871
Palestine	Asia	88	0.1	4.9	157,593	1812	5,101,416	4450	0.686
Cameroon	Africa	22	0.1	4.8	29,617	462	26,545,864	3365	0.556
Slovenia	Europe	162	0.1	4.7	163,235	3448	2,078,932	31,401	0.896
Cyprus	Europe	8	0.0	4.1	30,538	197	875,899	32,415	0.869
Egypt	Africa	366	0.2	4.0	164,282	9169	102,334,403	10,55	0.696
Czech Republic	Europe	614	0.1	3.9	964,66	15,944	10,708,982	32606	0.888
Jamaica	North America	13	0.1	3.8	15,435	344	2 961,161	8194	0.732
France minus IHU Méditerranée Infection *	*Europe*	*2794*	*0.1*	*3.8*	*3,135,908*	*73,968*	*-*	*-*	*-*
Serbia	Europe	146	0.0	3.7	390,637	3965	6,804,596	14,049	0.787
Italy	Europe	2974	0.1	3.4	2,515,507	87,381	60,461,828	35,22	0.880
India	Asia	4778	0.0	3.1	10,720,048	154,01	1,380,004,385	6427	0.640
North Macedonia	Europe	82	0.1	2.9	91,891	2831	2,083,380	13,111	0.757
Slovakia	Europe	122	0.1	2.8	243,427	4411	5,459,643	30,155	0.855
Russia	Europe	1820	0.0	2.6	3,752,548	70,533	145,934,460	24,766	0.816
Greece	Europe	141	0.1	2.5	154,796	5742	10,423,056	24,574	0.870
Kuwait	Asia	23	0.0	2.4	163,45	958	4,270,563	65,531	0.803
Hungary	Europe	278	0.1	2.3	363,45	12,291	9,660,350	26,778	0.838
Madagascar	Africa	6	0.0	2.2	18,743	279	27,691,019	1416	0.519
Belarus	Europe	35	0.0	2.1	242,851	1688	9,449,321	17,168	0.808
Turkey	Asia	493	0.0	1.9	2,457,118	25,605	84,339,067	25,129	0.791
Kazakhstan	Asia	53	0.0	1.7	231,716	3040	18,776,707	24,056	0.800
Montenegro	Europe	12	0.0	1.5	60,288	790	628,062	16,409	0.814
Morocco	Africa	122	0.0	1.5	469,139	8224	36,910,558	7485	0.667
Ecuador	South America	208	0.1	1.4	246	14,766	17,643,060	10,582	0.752
Argentina	South America	662	0.0	1.4	1,905,524	47,601	45,195,777	18,934	0.825
Belize	North America	4	0.0	1.3	11,845	298	397,621	7824	0.708
Myanmar	Asia	41	0.0	1.3	139,152	3103	54,409,794	5592	0.578
Poland	Europe	473	0.0	1.3	1,496,665	36,443	37,846,605	27,216	0.865
Tunisia	Africa	78	0.0	1.2	204,351	6508	11,818,618	10,849	0.735
Peru	South America	441	0.0	1.1	1,113,970	40,272	32,971,846	12,237	0.750
Brazil	South America	2414	0.0	1.1	9,058,687	221,547	212,559,409	14,103	0.759
Indonesia	Asia	313	0.0	1.1	1,037,993	29,331	273,523,621	11,189	0.694
Romania	Europe	191	0.0	1.1	721,513	18,105	19,237,682	23,313	0.811
Croatia	Europe	50	0.0	1.0	230,978	4943	4,105,268	22,67	0.831
Andorra	Europe	1	0.0	1.0	9779	100	77,265	-	0.858
Cuba	North America	2	0.0	1.0	24,105	208	11,326,616	-	0.777
Lebanon	Asia	23	0.0	0.9	293,157	2621	6,825,442	13,368	0.757
Georgia	Asia	26	0.0	0.8	256,287	3127	3,989,175	9745	0.780
Kosovo	Europe	12	0.0	0.8	58,988	1479	1,932,774	9796	-
Nepal	Asia	15	0.0	0.7	270,588	2020	29,136,808	2443	0.574
Bosnia and Herzegovina	Europe	33	0.0	0.7	121,194	4659	3,280,815	11,714	0.768
Algeria	Africa	18	0.0	0.6	106,61	2881	43,851,043	13,914	0.754
Guatemala	North America	32	0.0	0.6	157,595	5543	17,915,567	7424	0.650
Colombia	South America	290	0.0	0.5	2,067,575	52,913	50,882,884	13,255	0.747
Pakistan	Asia	58	0.0	0.5	541,031	11,56	220,892,331	5035	0.562
Mexico	North America	598	0.0	0.4	1,825,519	155,145	128,932,753	17,336	0.774
El Salvador	North America	6	0.0	0.4	53,989	1599	6,486,201	7292	0.674
Philippines	Asia	38	0.0	0.4	519,575	10,552	109,581,085	7599	0.699
Dominican Republic	North America	8	0.0	0.3	208,61	2603	10,847,904	14,601	0.736
Ukraine	Europe	67	0.0	0.3	1,247,674	23,469	43,733,759	7894	0.751
Zambia	Africa	2	0.0	0.3	50,319	705	18,383,956	3689	0.588
Bolivia	South America	27	0.0	0.3	210,726	10,226	11,673,029	6886	0.693
Moldova	Europe	9	0.0	0.3	158,309	3413	4,033,963	5190	0.700
Azerbaijan	Asia	8	0.0	0.3	229,793	3113	10,139,175	15,847	0.757
Venezuela	South America	3	0.0	0.3	125,364	1171	28,435,943	16,745	0.761
Iraq	Asia	31	0.0	0.2	617,202	13,024	40,222,503	15,664	0.685
Bulgaria	Europe	15	0.0	0.2	217,574	8973	6,948,445	18,563	0.813
Armenia	Asia	3	0.0	0.1	166,669	3067	2,963,234	8788	0.755
Albania	Europe	1	0.0	0.1	75,454	1350	2,877,800	11,803	0.785
Iran	Asia	36	0.0	0.1	1,398,841	5, 736	83,992,953	19,083	0.798
Saint Kitts and Nevis	North America	3	8.1	0.0	37	-	53,192	24,654	0.778
Cambodia	Asia	4	0.9	0.0	463	-	16,718,971	3645	0.582
Hong Kong	Asia	344	0.0	0.0	-	-	7,496,988	56,055	0.933
Slovakia	Europe	122	0.1	2.8	243,427	4411	5,459,643	30,155	0.855

* Our institute; GDP, gross domestic product; GDP per capita is in US dollars.

## Data Availability

Data are available from the GISAID database (https://www.gisaid.org/; accessed on 28 January 2021), from the “Our world in data” website (https://ourworldindata.org/; accessed on 28 January 2021), or from the corresponding author upon reasonable request.

## Supplementary Materials

The following are available online at https://www.mdpi.com/article/10.3390/v13050775/s1, Table S1: Top 100 laboratories that sequenced SARS-CoV-2 genomes, Table S2: Number of SARS-CoV-2 genomes in various sequence databases, and number of genomes per 100 SARS-CoV-2-diagnosed cases and per 100 SARS-CoV-2-associated deaths according to country.
