# Supplementary material for: Global Discrepancies between Numbers of Available SARS-CoV-2 Genomes and Human Development Indexes at Country Scales
Source: Viruses. 2021 Apr 28;13(5):775. doi: 10.3390/v13050775 (PMC8145975; doi:10.3390/v13050775)
Supplement: Supplementary file 1 [file viruses-13-00775-s001.zip › SupplTabS1_viruses-1153266.pdf]

Supplementary Table S1. Top 100 laboratories that sequenced SARS-CoV-2 genomes

Rank	Laboratory	Continent	Country	Region	Number of laboratories of the same country in the top 100	Number of genomes
1	Lighthouse Lab in Alderley Park	Europe	United Kingdom	England	21	**32 839**
2	Lighthouse Lab in Glasgow	Europe	United Kingdom	England	21	**22 378**
3	Lighthouse Lab in Milton Keynes	Europe	United Kingdom	England	21	**21 880**
4	Wales Specialist Virology Centre Sequencing lab: Pathogen Genomics Unit	Europe	United Kingdom	Wales	5	**16 321**
5	Lighthouse Lab in Cambridge	Europe	United Kingdom	England	21	**16 059**
6	Pathogen Genomics Center, National Institute of Infectious Diseases	Asia	Japan		3	**12 748**
7	Department of Virus and Microbiological Special Diagnostics, Statens Serum Institut, Copenhagen, Denmark	Europe	Denmark	Hovedstaden	11	**10 445**
8	Microbiological Diagnostic Unit - Public Health Laboratory (MDU-PHL)	Oceania	Australia	Victoria	2	**10 036**
9	Department of Pathology, University of Cambridge	Europe	United Kingdom	England	21	**8 505**
10	Houston Methodist Hospital	North America	USA	Texas, Houston	24	**7 850**
11	Respiratory Virus Unit, Microbiology Services Colindale, Public Health England	Europe	United Kingdom	England	21	**6 014**
12	Oxford Viromics, NDM, University of Oxford; Oxford University Hospitals; Basingstoke and North Hampshire Hospital	Europe	United Kingdom	England	21	**5 425**
13	Houston Methodist Hospital	North America	USA	Texas, Greater Houston Area	24	**5 085**
14	Liverpool Clinical Laboratories	Europe	United Kingdom	England	21	**4 687**
15	Department of Virus and Microbiological Special Diagnostics, Statens Serum Institut, Copenhagen, Denmark	Europe	Denmark	Midtjylland	11	**4 452**
16	Quadram Institute Bioscience	Europe	United Kingdom	England	21	**4 430**
17	Department of Virus and Microbiological Special Diagnostics, Statens Serum Institut, Copenhagen, Denmark	Europe	Denmark	Syddanmark	11	**4 160**
18	Virology Department, Sheffield Teaching Hospitals NHS Foundation Trust/Department of Infection, Immunity and Cardiovascular Disease, The Medical School, University of Sheffield	Europe	United Kingdom	England	21	**4 110**
19	West of Scotland Specialist Virology Centre, NHSGGC / MRC-University of Glasgow Centre for Virus Research	Europe	United Kingdom	Scotland	5	**4 005**
20	Lighthouse Lab in Glasgow	Europe	United Kingdom	Scotland	5	**3 875**
21	Utah Public Health Laboratory	North America	USA	Utah	24	**3 751**
22	Victorian Infectious Diseases Reference Laboratory (VIDRL)	Oceania	Australia	Victoria	2	**3 698**
23	Department of Virus and Microbiological Special Diagnostics, Statens Serum Institut, Denmark	Europe	Denmark	Hovedstaden	11	**3 682**
24	Department of Virus and Microbiological Special Diagnostics, Statens Serum Institut, Copenhagen, Denmark	Europe	Denmark	Sjaelland	11	**3 448**
25	University College London, Great Ormond Street Hospital for Children NHS Foundation Trust, Imperial College Healthcare NHS Trust	Europe	United Kingdom	England	21	**3 365**
26	PHE South West Regional Laboratory, National Infection Service	Europe	United Kingdom	England	21	**3 306**
27	Michigan Department of Health and Human Services, Bureau of Laboratories	North America	USA	Michigan	24	**3 104**
28	deCODE genetics	Europe	Iceland	Reykjavik	2	**2 915**
29	Wales Specialist Virology Centre	Europe	United Kingdom	Wales	5	**2 844**
30	Virology Department, Royal Infirmary of Edinburgh, NHS Lothian / School of Biological Sciences, University of Edinburgh / Institute of Genetics and Molecular Medicine, University of Edinburgh	Europe	United Kingdom	Scotland	5	**2 736**
31	Queens Medical Centre, Clinical Microbiology Department / DeepSeq Nottingham	Europe	United Kingdom	England	21	**2 681**
32	Florida Bureau of Public Health Laboratories	North America	USA	Florida	24	**2 655**
33	Department of Virus and Microbiological Special Diagnostics, Statens Serum Institut, Copenhagen, Denmark	Europe	Denmark	Nordjylland	11	**2 630**
34	UW Virology Lab	North America	USA	Washington	24	**2 600**
35	Northumbria University / South Tees Hospitals NHS Foundation Trust / North Cumbria Integrated Care NHS Foundation Trust / North Tees and Hartlepool NHS Foundation Trust / Newcastle Hospitals NHS Foundation Trust	Europe	United Kingdom	England	21	**2 518**
36	Centre for Enzyme Innovation, University of Portsmouth / Translational Research Laboratory, Portsmouth Hospitals NHS Trust	Europe	United Kingdom	England	21	**2 466**
37	Laboratoire national de santé, Microbiology, Virology	Europe	Luxembourg	Luxembourg	1	**2 054**
38	Washington State Department of Health	North America	USA	Washington, Yakima County	24	**1 950**
39	Lighthouse Lab in Alderley Park	Europe	United Kingdom	Wales	5	**1 916**
40	University of Wisconsin-Madison AIDS Vaccine Research Laboratories	North America	USA	Wisconsin, Dane County	24	**1 861**
41	Respiratory Virus Unit, National Infection Service, Public Health England	Europe	United Kingdom	England	21	**1 855**

Supplementary Table S1. Top 100 laboratories that sequenced SARS-CoV-2 genomes

Rank	Laboratory	Continent	Country	Region	Number of laboratories of the same country in the top 100	Number of genomes
42	University of Michigan Clinical Microbiology Laboratory	North America	USA	Michigan	24	**1 720**
43	Dutch COVID-19 response team	Europe	Netherlands	South Holland	6	**1 700**
44	Alberta Precision Labs (APL)	North America	Canada	Alberta	5	**1 674**
45	University of Exeter	Europe	United Kingdom	England	21	**1 651**
46	San Diego County Public Health Laboratory	North America	USA	California, San Diego	24	**1 610**
47	Regional Virus Laboratory, Belfast Health and Social Care Trust	Europe	United Kingdom	Northern Ireland	1	**1 594**
48	IHU Mediterranee Infection	Europe	France	Provence-Alpes-Côte d'Azur, Marseille	1	**1 585**
49	Laboratoire de santé publique du Québec	North America	Canada	Quebec	5	**1 540**
50	Department of Virus and Microbiological Special Diagnostics, Statens Serum Institut, Denmark	Europe	Denmark	Syddanmark	11	**1 393**
51	Department of Clinical Microbiology	Europe	Belgium	Liège	1	**1 353**
52	NHSGGC West of Scotland Specialist Virology Centre / MRC-University of Glasgow Centre for Virus Research	Europe	United Kingdom	Scotland	5	**1 350**
53	University of Birmingham	Europe	United Kingdom	England	21	**1 349**
54	Virginia DCLS	North America	USA	Virginia	24	**1 338**
55	Department of Virus and Microbiological Special Diagnostics, Statens Serum Institut, Denmark	Europe	Denmark	Midtjylland	11	**1 307**
56	Instituto Nacional de Saude (INSA)	Europe	Portugal	Portugal	1	**1 287**
57	Originating lab: Wales Specialist Virology Centre Sequencing lab: Pathogen Genomics Unit	Europe	United Kingdom	Wales	5	**1 268**
58	The National University Hospital of Iceland	Europe	Iceland	Reykjavik	2	**1 260**
59	National Public Health Laboratory, National Centre for Infectious Diseases	Asia	Singapore	Singapore, Singapore	1	**1 136**
60	Bioinformatics and Biostatistics Lab, Advanced Sequencing Facility	Europe	United Kingdom	England	21	**1 126**
61	Department of Virology and Immunology, University of Helsinki and Helsinki University Hospital, Huslab Finland	Europe	Finland	Uusimaa	1	**1 114**
62	AZ SPHL, Arizona Department of Health Services	North America	USA	Arizona	24	**1 109**
63	Quest Diagnostics	North America	USA	California	24	**1 101**
64	Group 42 (G42) Healthcare, Abu Dhabi, United Arab Emirates; Department of Health, The United Arab Emirates	Asia	United Arab Emirates	Abu Dhabi	1	**1 067**
65	Dutch COVID-19 response team	Europe	Netherlands	North Brabant	6	**1 061**
66	Santa Clara County Public Health Laboratory	North America	USA	California, Santa Clara County	24	**1 032**
67	Viollier AG	Europe	Switzerland	Bern	3	**980**
68	Minnesota Department of Health, Public Health Laboratory	North America	USA	Minnesota	24	**979**
69	NU-OMICS DNA Sequencing research facility, Northumbria University	Europe	United Kingdom	England	21	**953**
70	Public Health Ontario Laboratory	North America	Canada	Ontario	5	**944**
71	University Medical Center Hamburg Eppendorf	Europe	Germany	Hamburg	1	**942**
72	Viollier AG	Europe	Switzerland	Zürich	3	**921**
73	BCCDC Public Health Laboratory	North America	Canada	British Columbia	5	**920**
74	Massachusetts General Hospital	North America	USA	Massachusetts	24	**919**
75	Massachusetts State Public Health Laboratory	North America	USA	Massachusetts	24	**916**
76	Department of Virus and Microbiological Special Diagnostics, Statens Serum Institut, Denmark	Europe	Denmark	Sjaelland	11	**912**
77	Wyoming Public Health Laboratory	North America	USA	Wyoming	24	**899**
78	Dutch COVID-19 response team	Europe	Netherlands	Utrecht	6	**895**
79	Department of Virus and Microbiological Special Diagnostics, Statens Serum Institut, Denmark	Europe	Denmark	Nordjylland	11	**867**
80	Ginkgo Bioworks Clinical Laboratory	North America	USA	Utah	24	**845**
81	Hospital General Universitario Gregorio Marañón	Europe	Spain	Madrid, Madrid	1	**843**
82	New Mexico Department of Health Scientific Laboratory	North America	USA	New Mexico	24	**839**
83	Toronto Invasive Bacterial Diseases Network	North America	Canada	Ontario, Toronto	5	**829**
84	Dutch COVID-19 response team	Europe	Netherlands	Netherlands	6	**824**
85	TGen North	North America	USA	Arizona	24	**799**
86	Lighthouse Lab in Milton Keynes	Europe	United Kingdom	Wales	5	**785**

Supplementary Table S1. Top 100 laboratories that sequenced SARS-CoV-2 genomes

Rank	Laboratory	Continent	Country	Region	Number of laboratories of the same country in the top 100	Number of genomes
87	Division of Emerging Infectious Diseases, Bureau of Infectious Diseases Diagnosis Control, Korea Disease Control and Prevention Agency	Asia	South Korea	South Korea	2	**775**
88	Department of Infectious Diseases, Kobe Institute of Health	Asia	Japan	Japan	3	**763**
89	Virology Department, Royal Infirmary of Edinburgh, NHS Lothian / School of Biological Sciences, University of Edinburgh	Europe	United Kingdom	Scotland	5	**759**
90	LSUHS Emerging Viral Threat Laboratory	North America	USA	Louisiana, Caddo Parish	24	**755**
91	Dutch COVID-19 response team	Europe	Netherlands	Gelderland	6	**742**
92	University College London Hospital	Europe	United Kingdom	England	21	**740**
93	Division of Viral Diseases, Center for Laboratory Control of Infectious Diseases, Korea Centers for Diseases Control and Prevention	Asia	South Korea	South Korea	2	**726**
94	Dutch COVID-19 response team	Europe	Netherlands	North Holland	6	**715**
95	Viollier AG	Europe	Switzerland	Basel-Land	3	**700**
96	Fukuoka Institute of Health and Environmental Sciences	Asia	Japan	Japan	3	**694**
97	Sonora Quest Laboratories, Laboratory Sciences of Arizona	North America	USA	Arizona	24	**686**
98	Department of Virus and Microbiological Special Diagnostics, Statens Serum Institut, Copenhagen, Denmark	Europe	Denmark	Denmark	11	**642**
99	OHSU Lab Services Molecular Microbiology Lab	North America	USA	Oregon, Washington County OR	24	**640**
100	Istituto Zooprofilattico Sperimentale del Mezzogiorno	Europe	Italy	Campania	1	**625**
