# Supplementary material for: Global Discrepancies between Numbers of Available SARS-CoV-2 Genomes and Human Development Indexes at Country Scales
Source: Viruses. 2021 Apr 28;13(5):775. doi: 10.3390/v13050775 (PMC8145975; doi:10.3390/v13050775)
Supplement: Supplementary file 1 [file viruses-13-00775-s001.zip › SupplTabS2_viruses-1153266.pdf]

Country	Continent/ region	Number of cases	**Number of deaths**	Human development index	Number of genomes (GISAID)	Number of genomes (NCBI - Nucleotide)	Number of genomes (NCBI - Sequence Read Archive (SRA))	Number of genomes (Total NCBI)	Number of genomes (EMBL-EBI)	Number of genomes (China NCB)	**Number of genomes per 100 deaths (GISAID)**	**Number of genomes per 100 deaths (Total NCBI)**	**Number of genomes per 100 deaths (EMBL-EBI)**	**Number of genomes per 100 deaths (China NCB)**
Denmark	Europe	197 892	**2 072**	0,929	34 819	12	0	12	12	34 506	**1 680**	**1,0**	**1,0**	**1 665**
United Kingdom	Europe	3 754 448	**103 324**	0,922	192 556	743	1519	2262	2242	190 140	**186**	**2,0**	**2,0**	**184**
South Korea	Asia	77 395	**1 399**	0,903	1 631	20	16	36	19	1 631	**117**	**3,0**	**1,0**	**117**
Netherlands	Europe	979 702	**13 925**	0,931	7 422	1545	1557	3102	1546	5 874	**53**	**22**	**11**	**42**
Canada	North America	770 433	**19 659**	0,926	8 613	18	832	850	18	8 113	**44**	**4,0**	**0,0**	**41**
Ghana	Africa	63 883	**390**	0,592	114	77	0	77	240	144	**29**	**20**	**62**	**37**
USA	North America	25 766 681	**433 196**	0,924	89 814	32792	25685	58477	61437	88 928	**21**	**13**	**14**	**21**
China	Asia	99 746	**4 813**	0,752	949	239	599	838	241	2 081	**20**	**17**	**5,0**	**43**
Nigeria	Africa	127 024	**1 547**	0,532	290	4	0	4	4	261	**19**	**0,0**	**0,0**	**17**
Germany	Europe	2 194 562	**55 883**	0,936	4 582	128	523	651	99	4 536	**8,2**	**1,0**	**0,0**	**8,1**
South Africa	Africa	1 437 798	**43 105**	0,699	3 062	1	0	1	518	2 836	**7,1**	**0,0**	**1,0**	**6,6**
France	Europe	3 166 145	**74 601**	0,901	4 379	93	145	238	96	3 813	**5,9**	**0,0**	**0,0**	**5,1**
Gabon	Africa	10 536	**68**	0,702	4	77	0	77	11	12	**5,9**	**113**	**16**	**18**
Egypt	Africa	164 282	**9 169**	0,696	366	477	3	480	522	602	**4,0**	**5,0**	**6,0**	**6,6**
Italy	Europe	2 515 507	**87 381**	0,88	2 974	209	128	337	217	2 933	**3,4**	**0,0**	**0,0**	**3,4**
India	Asia	10 720 048	**154 010**	0,64	4 778	704	2473	3177	771	2 933	**3,1**	**2,0**	**1,0**	**1,9**
Tunisia	Africa	204 351	**6 508**	0,735	78	71	0	71	72	123	**1,2**	**1,0**	**1,0**	**1,9**

National Center for Biotechnology Information (NCBI) - Nucleotide: https://www.ncbi.nlm.nih.gov/
National Center for Biotechnology Information (NCBI) - Sequence Read Archive (SRA): https://www.ncbi.nlm.nih.gov/sra/
European Bioinformatics Institute (EMBL-EBI): https://covid-19.ensembl.org/index.html
China National Center for Bioinformation (CNCB): https://bigd.big.ac.cn/ncov/
